# N-((1-(4-Fluorophenyl)-1H-1,2,3-triazol-4-yl)methyl)-2-methylene-3-oxo-olean-12-en-28-amide Induces Apoptosis in Human Breast Cancer Cells by Stimulating Oxidative Stress and Inhibiting the Notch-Akt Signaling Pathway

**DOI:** 10.1155/2022/8123120

**Published:** 2022-04-27

**Authors:** Xiaorui Li, Shisheng Wang, Ning Deng, Xiangyu Guo, Meiyi Fu, Yiwen Ma, Tao Sun

**Affiliations:** ^1^Cancer Hospital of Dalian University of Technology Liaoning Cancer Hospital & Institute, Shenyang, China; ^2^College of Pharmaceutical Science and Technology, Dalian University of Technology, Dalian, China

## Abstract

Breast cancer is of the leading causes of cancer-related deaths and the most frequently diagnosed cancer among females worldwide. Despite advancements in breast cancer therapy, the disease eventually progresses in most patients because of de novo or secondary resistance. Thus, discovering novel drugs with high effectiveness and low toxicity for systemic therapy is essential. In this study, we investigated whether a new oleanolic derivative N-((1-(4-fluorophenyl)-1H-1,2,3-triazol-4-yl)methyl)-2-methylene-3-oxo-olean-12-en-28-amide (ZQL-4c) exhibits potential anticancer effects against breast cancer. We determined that ZQL-4c strongly inhibited cell proliferation and invasion and induced G2/M phase arrest and apoptosis in breast cancer cells. We then found that ZQL-4c induced the production of reactive oxygen species (ROS). We then found that ZQL-4c significantly inhibited Notch-AKT signaling pathways that are related to oxidative stress. Taken together, this study is the first to show that ZQL-4c can significantly suppress the growth and invasion of breast cancer by blocking Notch-Akt signaling pathways, which are mainly regulated by ROS-mediated oxidative stress. Thus, ZQL-4c might be considered a novel and potential anticancer drug for breast cancer treatment.

## 1. Introduction

Breast cancer is the most frequently diagnosed cancer among women worldwide, accounting for 31% of total cancer cases. Simultaneously, it has also become one of the leading causes of cancer-related deaths, accounting for 15% of all cancer-related deaths according to the 2022 global cancer statistics [1]. Over the past decades, conventional therapies such as surgery, chemotherapy, radiotherapy, endocrine therapy, and targeted therapy have significantly reduced the risk of disease recurrence and death in patients with breast cancer [2–10]. Although oncological outcomes continue to improve, patients with the most advanced breast cancer ultimately die of the disease because of resistance to therapy [11, 12]. Therefore, discovering effective and safe drugs is essential to treat breast cancer.

Phytochemicals with antioxidant, anti-inflammatory, and immunomodulatory activities are commonly used as alternative or complementary therapies for cancer [13, 14]. Oleanolic acid (OA) is one of the most common phytochemicals and is present in nature as a free acid or as an aglycone of triterpenoid saponins [15]. OA exhibits antitumor activities against several neoplasms, including hepatocellular cancer [16], hematological malignancies [17], lung [18], ovarian [19], pancreatic [20], skin cancer [21], glioblastoma [22], and breast cancer [23]. Despite its effectiveness, research and clinical applications of OA are limited because of its poor water solubility. The potential mechanism underlying the antibreast cancer activity of OA and its derivatives is unclear.

In our previous study, more than 50 OA derivatives were synthesized, and their antitumor activities were examined against cancer cells. One of these OA derivatives, N-((1-(4-fluorophenyl)-1H-1,2,3-triazol-4-yl)methyl)-2-methylene-3-oxo-olean-12-en-28-amide (ZQL-4c), exhibits anticancer effect against breast cancer. In the present study, the anticancer activity and underlying molecular mechanisms of ZQL-4c in various types of breast cancer were studied. We found that ZQL-4c induces apoptosis in human breast cancer cells by stimulating oxidative stress and suppressing the Notch-AKT signaling pathway. These results indicated that ZQL-4c might be considered a promising drug for breast cancer.

## 2. Materials and Methods

### 2.1. Synthesis of OA Derivatives

**Figure 1:**
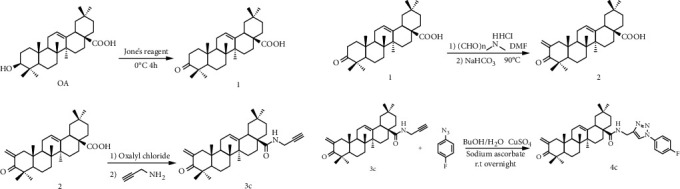
Synthesis of the OA derivative N-((1-(4-fluorophenyl)-1H-1,2,3-triazol-4-yl)methyl)-2-methylene-3-oxo-olean-12-en-28-amide (ZQL-4c.

The OA derivative ZQL-4c was synthesized by Professor Shisheng Wang from the Dalian University of Technology (Figure 1).

### 2.2. Chemical Reagents and Antibodies

ZQL-4c was dissolved in DMSO (D2650; Sigma, USA). A ZQL-4c stock solution of 8 mM was stored at −80°C. Mouse anti-*β*-actin (1 : 5000 dilution), rabbit anti-mTOR (1 : 1000 dilution), rabbit anti-p-mTOR (1 : 1000 dilution), rabbit anti-AKT (1 : 1000 dilution), rabbit anti-p-AKT (1 : 1000 dilution), rabbit anti-JAK2 (1 : 1000 dilution), rabbit anti-p-JAK2 (1 : 1000 dilution), rabbit anti-STAT3 (1 : 1000 dilution), rabbit anti-p-STAT3 (1 : 1000 dilution), rabbit anti-cleaved-caspase3 (1 : 1000 dilution), anti-cleaved-caspase7 (1 : 1000 dilution), anti-cleaved-caspase9 (1 : 1000 dilution), anti-p53 (1 : 1000 dilution), anti-BAX (1 : 1000 dilution), anti-BCL-2 (1 : 1000 dilution), anti-cyclin B1 (1 : 1000 dilution), anti-cyclin D1 (1 : 1000 dilution), anti-p21 (1 : 1000 dilution), anti-p27 (1 : 1000 dilution), Notch1 (1 : 1000 dilution), and Notch2 (1 : 1000 dilution) were purchased from CST company (Danvers, MA, USA).

### 2.3. Cell Culture

The human breast cancer MCF-7, MDA-MB-231, and SK-BR-3 cell lines were purchased from ATCC (ATCC; Rockville, MD). MCF-7 cells were cultured in MEM (L110KJ; Basal Media, China) with 10% fetal bovine serum (FBS; 10439024; Thermo Fisher Scientific, Inc.), 1% nonessential amino acids (11140050; Thermo Fisher Scientific, Inc.), and 1 mM sodium pyruvate (11360070; Thermo Fisher Scientific, Inc.) at 37°C with 5% CO_2_. MDA-MB-231 and SK-BR-3 cells were cultivated in DMEM (12800–058; Thermo Fisher Scientific, Inc.) with 10% FBS.

### 2.4. Cell Viability Assay

We evaluated cell viability using a Cell Counting Kit-8 (CCK8) assay kit (CK04; Dojindo Laboratories; Japan). MCF-7, MDA-MB-231, and SK-BR-3 cells (5 × 10^3^ cells/well) were seeded in 96-well plates and incubated with ZQL-4c (0, 0.4, 0.8, 1.0, 2.0, 4.0, and 8.0 *μ*mol/L) for 24 h and 48 h. DMSO at the same concentration was used as the control. After treatment, CCK8 was added to each well (10 *μ*L of CCK8 substrate and 90 *μ*L of medium) and incubated at 37°C for 2 h. The optical density (OD) was measured using a microplate reader at 450 nm (Multiskan MK3; Pioneer Co-operative UK Ltd.). The cell viability after ZQL-4c treatment was calculated using IBM SPSS Statistics 19 as follows: (OD experiment − OD background)/(OD negative − OD background) × 100.

### 2.5. Apoptosis Analysis

We detected apoptosis by Annexin-V FITC/PI staining. MCF-7, MDA-MB-231, and SK-BR-3 cells were seeded in 6-well plates (5 × 10^5^ cells/well) and treated with ZQL-4c (0, 0.4, 0.8, and 1.6 *μ*mol/L) for 24 h. After incubation, the cells were collected and incubated at room temperature with 5 *μ*L of Annexin-V/FITC (C1052; Beyotime Institute of Biotechnology, China) in the binding buffer in the dark for 20 min. PI solution was then added and incubated for 10 min. The cells were analyzed by fluorescence-activated cell sorting (FACS) using a flow cytometer (BD FACSAria II; BD Co.).

### 2.6. Cell Cycle Analysis

MCF-7, MDA-MB-231, and SK-BR-3 cells cultured in 6-well plates (5 × 10^5^ cells/well) were synchronized by serum starvation overnight and treated with ZQL-4c (0, 0.4, 0.8, and 1.6 *μ*mol/L) for 24 h. After incubation, the cells were fixed with 70% ethanol at 4°C overnight and resuspended in ice-cold phosphate-buffered saline (PBS). Subsequently, the cell cycle solution (C1052; Beyotime Institute of Biotechnology) containing 50 *μ*g/mL PI and 20 *μ*g/mL RNase A was added to cells, followed by incubation in the dark for 30 min. A flow cytometer (BD FACSAria II; BD Co.) was used to determine the cell cycle distribution of the cells.

### 2.7. Wound Healing Assay

MCF-7, MDA-MB-231, and SK-BR-3 cells were harvested and seeded in 6-well plates (5 × 10^5^ cells/well). The cells were then treated with ZQL-4c (0, 0.4, 0.8, and 1.6 *μ*mol/L) for 24 h. Thereafter, we used a sterile 10 *μ*L pipette tip to produce a scratch and removed the drifting cells by washing with PBS three times. After washing, we observed the scratches using an inverted phase-contrast microscope (LV-150N; Nikon Corporation). The wound surface area was quantified by Image J software.

### 2.8. ROS Detection Assay

The detection of intracellular ROS levels was performed by 2′-7′dichlorofluorescin diacetate (DCFH-DA). MCF-7, MDA-MB-231, and SK-BR-3 cells were seeded in 6-well plates (3 × 10^5^ cells/well) and then treated with ZQL-4c (0, 0.4, 0.8, and 1.6 *μ*mol/L) for 24 h. Thereafter, the cells were washed with PBS twice and then incubated with DCFH-DA (10 uM) for 20 min at 37°C in the dark. We then assessed ROS levels using fluorescence microscopy (Leica, Wetzlar, Germany) and flow cytometry (BD FACSAria II; BD Co.).

### 2.9. Western Blotting

MCF-7, MDA-MB-231, and SK-BR-3 cells were cultured in 6-well plates (1 × 10^6^ cells/well) and incubated with ZQL-4c (0, 0.4, 0.8, and 1.6 *μ*mol/L) for 24 h. We used protein extraction kits (78835; Thermo Fisher Scientific, Inc.) to collect proteins from the cells and BCA protein assay kits to determine protein concentrations. Thereafter, cytoplasmic protein extracts were reconstituted in loading buffer and boiled for 5 min. Proteins (20–50 *μ*g/sample) were separated by electrophoresis in 8–12% sodium dodecyl sulfate–polyacrylamide gel electrophoresis and transferred onto polyvinylidene difluoride membranes. The membranes were incubated in 5% (*w*/*v*) nonfat milk for 1 h at room temperature and then incubated overnight at 4°C on a shaker with specific primary antibodies. Subsequently, we incubated the membranes with secondary antibodies for 2 h at room temperature. The blots were developed by chemiluminescence and detected using an ImageQuant Analyzer (ImageQuant LAS 4000, GE Healthcare).

### 2.10. Statistical Analysis

Data are expressed as the mean ± standard error of the mean of at least three independent experiments. One-way analysis of variance was used as the statistical analysis method, and Student's *t*-test was used to compare two groups. SPSS 19.0 for Windows and GraphPad Prism version 8.02 were used to perform all statistical analyses. A *P* value of < 0.05 was considered statistically significant.

## 3. Results

### 3.1. ZQL-4c Significantly Inhibits the Growth of Breast Cancer Cells

**Figure 2:**
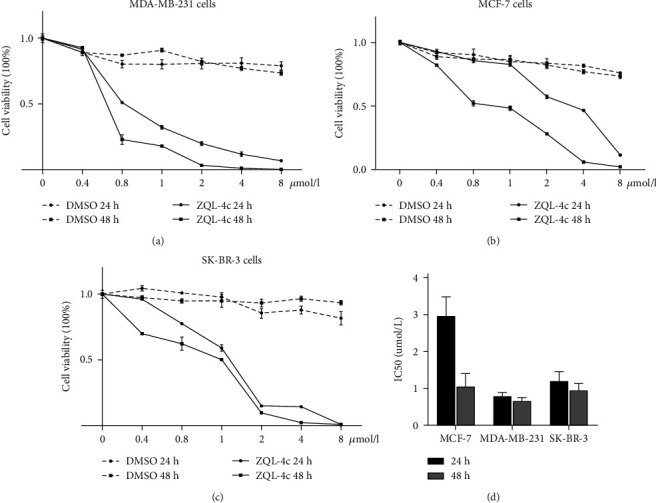
ZQL-4c significantly inhibited the growth of breast cancer cells. (a)–(c) MCF-7, MDA-MB-231, and SK-BR-3 cells were treated with various concentrations of ZQL-4c for increasing times. Line graphs showed the percentage of viable MCF-7, MDA-MB-231, and SK-BR-3 cells after treatment with 0, 0.4, 0.8, 1.0, 2.0, 4.0, and 8.0 *μ*mol/L ZQL-4c for 24 and 48 h, as determined by the CCK8 assay. Data are expressed as the mean ± standard error of the mean of three independent experiments performed in triplicate (*n* = 3; ^∗^*P* < 0.05). (d) Histogram showing the IC_50_ values (24 h and 48 h) of ZQL-4c in MCF-7, MDA-MB-231, and SK-BR-3 cells.

To investigate the cytotoxic effects of ZQL-4c, we treated human breast cancer cells (MCF-7, MDA-MB-231, and SK-BR-3) with different concentrations of ZQL-4c (0, 0.4, 0.8, 1.0, 2.0, 4.0, and 8.0 *μ*mol/L) for increasing times (24 h and 48 h) and analyzed them by performing the CCK8 assay. The analysis showed that ZQL-4c exhibited cytotoxic activity against MCF-7, MDA-MB-231, and SK-BR-3 cells in a dose- and time-dependent manner. In contrast, the inhibitory effects of the DMSO control group were slight (Figures 2(a)–2(c)). The IC_50_ values were 2.96 *μ*mol/L and 1.06 *μ*mol/L for MCF-7 cells, 0.80 *μ*mol/L and 0.67 *μ*mol/L for MDA-MB-231 cells, and 1.21 *μ*mol/L and 0.79 *μ*mol/L for SK-BR-3 cells after 24 h and 48 h of ZQL-4c treatment, respectively (Figure 2(d)). Altogether, ZQL-4c significantly inhibited the proliferation and growth of breast cancer cells.

### 3.2. ZQL-4c Induces G2/M Phase Arrest in Breast Cancer Cells

**Figure 3:**
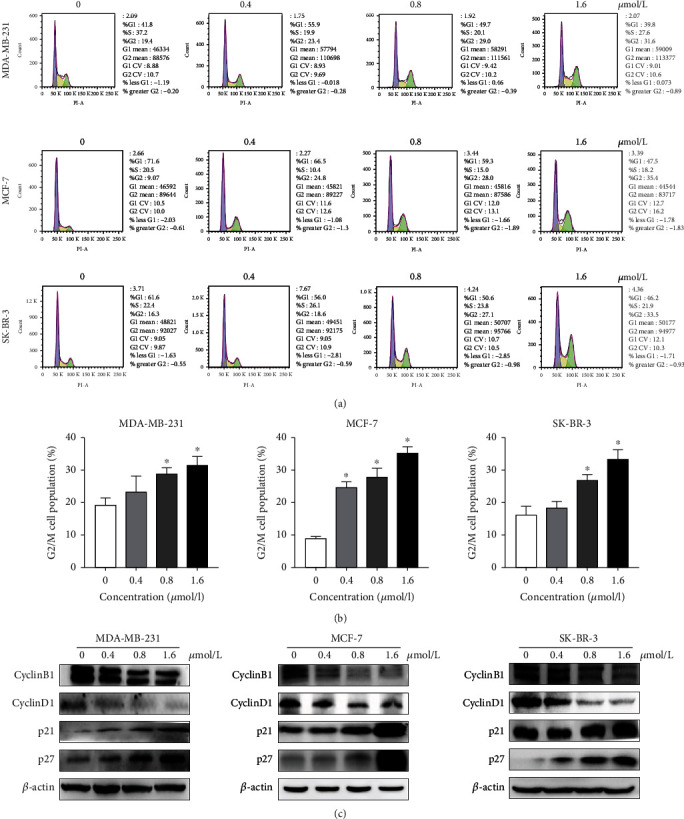
ZQL-4c induces G2/M phase arrest in breast cancer cells. MCF-7, MDA-MB-231, and SK-BR-3 cells were treated with 0, 0.4, 0.8, and 1.6 *μ*mol/L ZQL-4c for 24 h. (a) Cells were fixed and stained with PI and analyzed by flow cytometry. (b) The percentage of cells in the G2/M phase is shown in the histogram. Data are expressed as the mean ± standard error of the mean of three independent experiments performed in triplicate (*n* = 3; ^∗^*P* < 0.05). (c) The protein levels of cyclin B1, cyclin D1, p21, and p27 were detected by western blotting. Actin was used as a loading control.

To evaluate whether ZQL-4c induces cell cycle arrest in breast cancer cells, we performed cell cycle analysis by flow cytometry and found that the G2/M phase was enhanced in three breast cancer cells after ZQL-4c treatment in a dose-dependent manner. As shown in Figures 3(a) and 3(b), the G2/M population of MCF-7 cells treated with 0, 0.4, 0.8, and 1.6 *μ*mol/L of ZQL-4c for 24 h was 9.1%, 24.8%, 28.0%, and 35.4%, respectively; the G2/M population of MDA-MB-231 cells was 19.4%, 23.4%, 29%, and 31.6%, respectively; the G2/M population of SK-BR-3 cells was 16.3%, 18.6%, 27.1%, and 33.5%, respectively. Furthermore, the proteins cyclinD1, cyclinB1, p21, and p27 that regulate the G2/M phase were assessed by western blotting. As shown in Figure 3(c), ZQL-4c treatment decreased the protein levels of cyclinD1 and B1, whereas increased the protein levels of p21 and p27 in a dose-dependent manner in MCF-7, MDA-MB-231, and SK-BR-3 cells. These results showed that breast cancer cells are capable of being induced by ZQL-4c and stalled in the G2/M phase.

### 3.3. ZQL-4c Induces Apoptosis in Breast Cancer Cells

**Figure 4:**
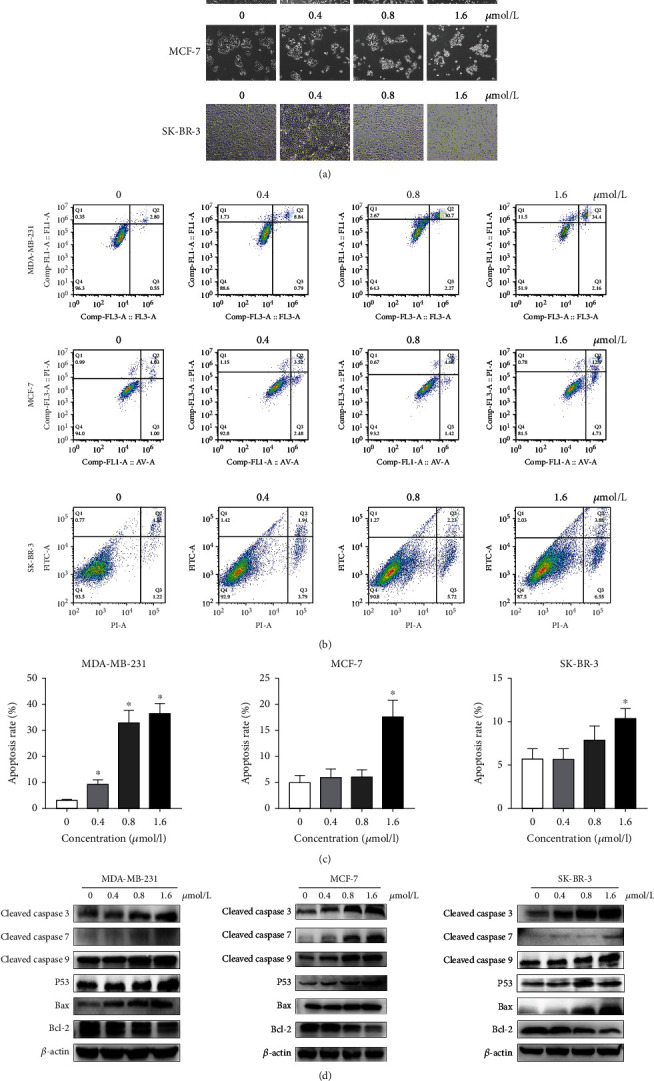
ZQL-4c induces apoptosis in breast cancer cells. MCF-7, MDA-MB-231, and SK-BR-3 cells were treated with 0, 0.4, 0.8, and 1.6 *μ*mol/L ZQL-4c for 24 h. (a) Light microscopy showed apoptosis-related changes induced by ZQL-4c. (b) Cells were stained with annexin V/PI and analyzed by flow cytometry. (c) The percentage of apoptotic cells is shown in the histogram. Data are expressed as the mean ± standard mean of the error of three independent experiments performed in triplicate (*n* = 3; ^∗^*P* < 0.05). (d) The protein levels of cleaved caspase-3,-7,-9, p53, Bax, and Bcl-2 were detected by western blotting. Actin was used as a loading control.

To assess whether apoptosis contributed to the inhibition of breast cancer cell growth induced by ZQL-4c, we performed annexin V/PI staining assays. The results showed that the apoptotic cell death rate increased as the ZQL-4c concentration increased. As shown in Figures 4(b) and 4(c), the total apoptotic cell rates of MCF-7 cells treated with ZQL-4c (0, 0.4, 0.8, and 1.6 *μ*mol/L) were 5.0%, 6.0%, 6.1%, and 17.6%, respectively; the total apoptotic cell rates of MDA-MB-231 cells were 3.4%, 9.6%, 32.97%, and 36.56%, respectively; the total apoptotic cell rates of SK-BR-3 cells were 5.7%, 5.7%, 8.0%, and 10.4%, respectively. In addition, we used light electron microscopy to observe ZQL-4c-dependent changes in cell morphology. As shown in Figure 4(a), we found that the cells shrank round and floated after ZQL-4c treatment. These results indicated that ZQL-4c induced apoptosis in breast cancer cells.

### 3.4. ZQL-4c Activates Caspase-Dependent Apoptotic Signaling Pathways

To identify the apoptotic signaling pathways of breast cancer cells, we performed western blotting and detected the levels of proteins associated with apoptosis after ZQL-4c treatment in MCF-7, MDA-MB-231, and SK-BR-3 cells. As shown in Figure 4(d), after treatment with 0, 0.4, 0.8, and 1.6 *μ*mol/L of ZQL-4c for 24 h, we observed an enhanced level of cleaved caspase -3, -7, -9, and p53. The apoptotic pathway that involves Bcl-2 family members can activate the caspase cascade. Therefore, Bax and Bcl-2 were investigated in this study. As shown in Figure 4(d), the Bax level was increased, and the Bcl-2 level was decreased after ZQL-4c treatment. These results suggested ZQL-4c treatment-induced apoptosis via intrinsic pathways in breast cancer cells.

### 3.5. ZQL-4c Inhibits Cell Migration and Invasion in Breast Cancer Cells

**Figure 5:**
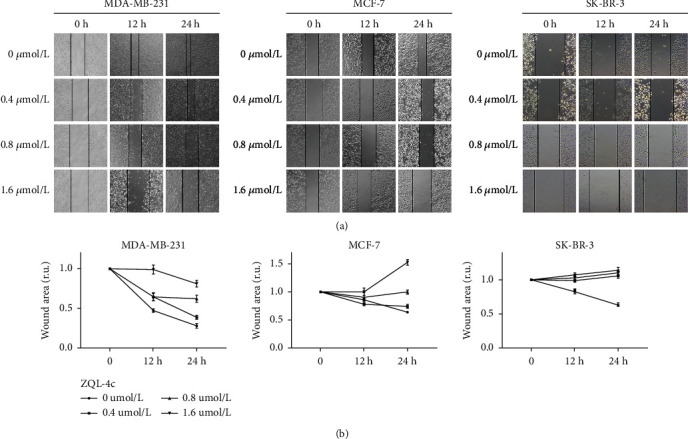
ZQL-4c inhibits cell migration and invasion in breast cancer cells. MCF-7, MDA-MB-231, and SK-BR-3 cells were treated with 0, 0.4, 0.8, and 1.6 *μ*mol/L ZQL-4c for 24 h. (a) A pipette tip was used to scratch the cultured cells and the width of the scratch was measured. Untreated cancer cultures of each cell line were used as negative controls. (b) The wound surface area was quantified by Image J software. Data are expressed as the mean ± standard error of the mean of three independent experiments performed in triplicate (*n* = 3; ^∗^*P* < 0.05).

To determine the effect of ZQL-4c on the migration of breast cancer cells, we performed a wound assay. To generate wounds, we scratched the cells using pipette tips and measured the width of the scratches after incubation with 0, 0.4, 0.8, and 1.6 *μ*mol/L of ZQL-4c in MCF-7, MDA-MB-231, and SK-BR-3 cells for 12 and 24 h. We also used cell lines that were not treated with ZQL-4c as negative controls. As shown in Figure 5, the relative scratch widths were significantly greater than that of the negative control after 24 h of ZQL-4c treatment in a dose-dependent manner. These results suggested that ZQL-4c treatment inhibited cell migration and invasion in breast cancer cells.

### 3.6. ZQL-4c Induces ROS Production in Breast Cancer Cells

**Figure 6:**
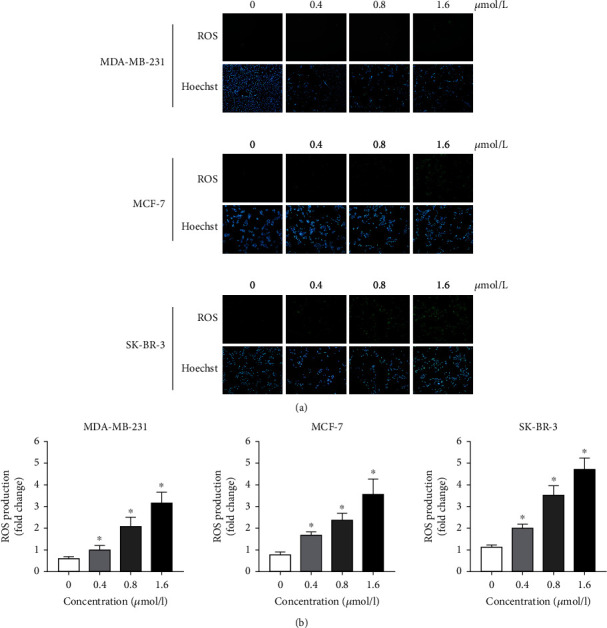
ZQL-4c induces reactive oxygen species production in breast cancer cells. MCF-7, MDA-MB-231, and SK-BR-3 cells were treated with 0, 0.4, 0.8, and 1.6 *μ*mol/L ZQL-4c for 24 h. Cells were loaded with 10 *μ*mol/L DCFH-DA for 20 min. The nuclei were stained with Hoechst 33342. The level of ROS production was detected by fluorescence microscopy (a) and flow cytometry (b).

To investigate whether oxidative stress played a pivotal role in ZQL-4c activity in breast cancer cells, we performed a DCFH-DA assay. As shown in Figures 6(a) and 6(b), we observed an increase in ROS production in a dose-dependent manner after ZQL-4c treatment by fluorescence microscopy and flow cytometry, indicating that ZQL-4c induced ROS-mediated oxidative stress in breast cancer cells.

### 3.7. ZQL-4c Suppresses the Notch and Akt Signaling Pathways in Breast Cancer Cells

**Figure 7:**
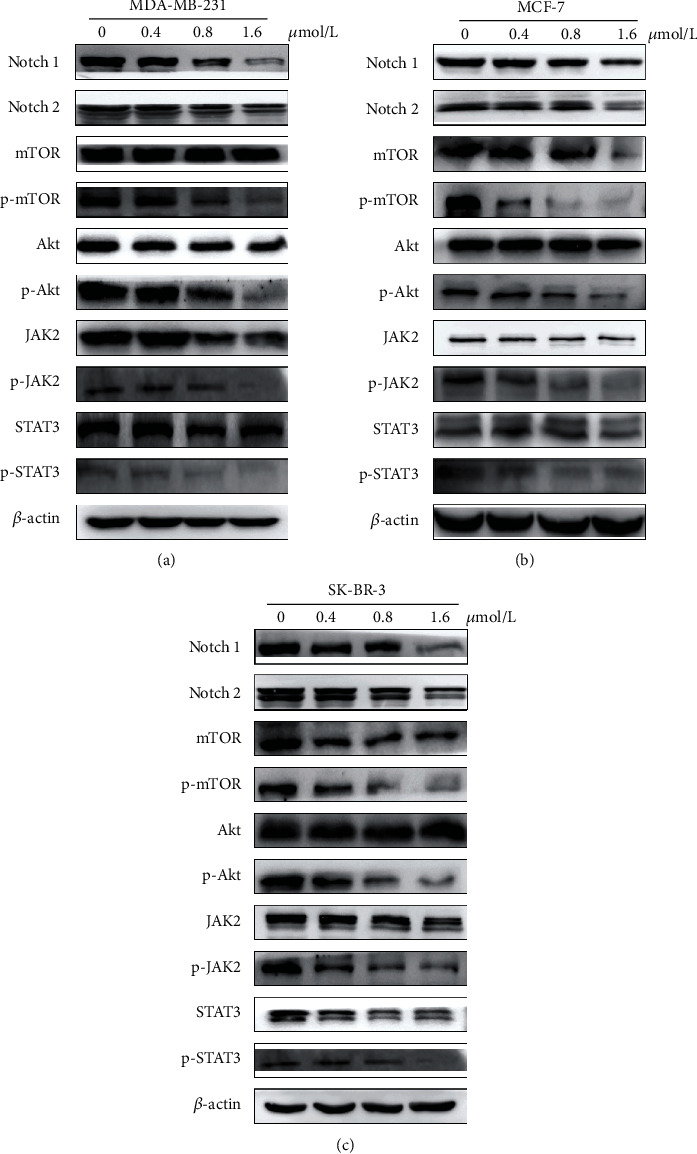
ZQL-4c suppresses Notch-Akt signaling pathways in breast cancer cells. MCF-7, MDA-MB-231, and SK-BR-3 cells were treated with 0, 0.4, 0.8, and 1.6 *μ*mol/L ZQL-4c for 24 h. (a)–(c) The protein levels of Notch1, Notch2, mTOR, phospho-mTOR, AKT, phospho-AKT, JAK2, phospho-JAK2, STAT3, and phospho-STAT3 were detected by western blotting. Actin was used as a loading control.

Notch and Akt pathways play pivotal roles in cancer cell proliferation and ROS production. To elucidate the mechanism of ZQL-4c effect on cell growth and ROS production, we investigated whether Notch and AKT pathways were regulated by ZQL-4c. As shown in Figures 7(a)–7(c), western blotting showed that ZQL-4c significantly reduced the expression of Notch1, Notch2, phospho-AKT, phospho-mTOR, phospho-STAT3, and phospho-JAK2 in a dose-dependent manner, with no marked change in AKT, mTOR, STAT3, and JAK2 levels. Thus, ZQL-4c inhibited Notch-AKT signaling pathways.

## 4. Discussion

OA, also known as 3*β*-hydroxyolean-12-en-28-oic acid, which belongs to the Oleaceae family, is a bioactive pentacyclic triterpenoid and can be isolated from >1,600 plant species [24–26]. OA exhibits antitumor activity against several neoplasms, including hepatocellular cancer, hematological malignancies, lung cancer, ovarian cancer, pancreatic cancer, skin cancer, glioblastoma, and breast cancer. Several studies have confirmed that OA exhibits a relatively weak antitumor activity against breast cancer because of poor water solubility [27]. Therefore, structural modifications may be a feasible strategy to improve the anticancer activity of OA by enhancing water solubility. In our previous study, more than 50 OA derivatives were synthesized and their anticancer activities were examined in various cancer cells. ZQL-4c is one of these OA derivatives with the chemical modification of A ring/C 28 and exhibits an anticancer effect on breast cancer. In this study, ZQL-4c significantly inhibited the proliferation and growth of breast cancer cells in a dose- and time-dependent manner. The IC_50_ values of ZQL-4c were about 0.7-3 *μ*mol/L, which were higher than those of OA. Among the three types of breast cancer cells, ZQL-4c exhibited the highest inhibitory effect on MDA-MB-231 cells (IC_50_: 0.80 *μ*mol/L for 24 h, 0.67 *μ*mol/L for 48 h), indicating that ZQL-4c was slightly selective against triple-negative breast cancer.

Apoptosis and cell cycle arrests play important roles in the destruction of undesired cells that are under the circumstance of pathology or aging [28]. In this study, various apoptotic assays were performed to identify the apoptotic effects of ZQL-4c on breast cancer cells. Morphology and flow cytometry data indicated that ZQL-4c inhibited the growth of breast cancer cells effectively via apoptosis. For elucidating the mechanism of apoptosis, western blotting showed that ZQL-4c stimulated caspase-3,-7, and-9 in three types of breast cancer cells, which are the key initiators of caspase cascades in apoptotic cells. The apoptotic pathway, which involves Bcl-2 family members, can activate the caspase cascade. Our results showed that the Bax level was increased, whereas the Bcl-2 level was decreased after ZQL-4c treatment, indicating that ZQL-4c treatment-induced apoptosis via intrinsic pathways. Meanwhile, the results of cell cycle analysis showed that G2/M cell cycle arrest was induced in breast cancer cells as ZQL-4c concentration increased, and the low levels of cell cycle proteins D1 and B1 and high levels of p21 and p27 were also observed.

ROS are potent stimulators of apoptosis and can activate the intrinsic mitochondrial pathway, the extrinsic death receptor pathway, and the endoplasmic reticulum stress pathway [29]. ROS stimulate events that lead to the loss of inner mitochondrial membrane permeability and control of the mitochondrial permeability transition pore complex, thus, disrupting membrane potential, releasing cytochrome c, and activating caspase-3, -7, and -9 [30, 31]. We observed that ZQL-4c induced ROS production in a dose-dependent manner, thus, inducing apoptosis with the upregulation of cleaved caspase-3, -7, and -9 in all types of breast cancer cells. These results suggested that ROS markedly contributed to the anticancer effect of ZQL-4c in breast cancer cells.

**Figure 8:**
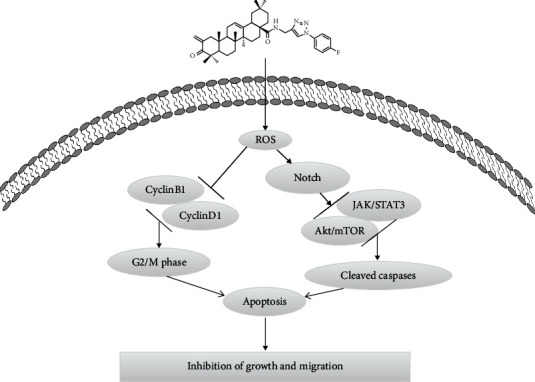
Proposed model for ZQL-4c-induced cell death in breast cancer cells.

Notch signaling is involved in many aspects of cancer biology, such as angiogenesis, tumor immunity, the maintenance of cancer stem-like cells, and oxidative stress [32, 33]. In addition, studies have reported an important role of Notch in regulating AKT signaling [34]. In this study, to investigate the mechanism of ROS upregulation after ZQL-4c treatment, we detected Notch-AKT signaling pathways. Our results showed that ZQL-4c significantly reduced the expression of Notch1 and Notch2. Moreover, ZQL-4c significantly decreased the expression of phospho-AKT and phospho-mTOR. Besides, ZQL-4c significantly inhibited the expression of phospho-STAT3 and phospho-JAK2 in three types of cancer cells. Thus, ZQL-4c induces ROS-mediated oxidative stress by inhibiting Notch-Akt signaling pathways (Figure 8).

Altogether, ZQL-4c can significantly suppress the growth and invasion of breast cancer by blocking Notch-Akt signaling pathways that are mainly regulated by ROS-mediated oxidative stress. Our findings showed that ZQL-4c can be developed as a novel and potential anticancer drug for breast cancer treatment.

## Data Availability

The raw/processed data required to reproduce these findings cannot be shared at this time as the data also forms part of an ongoing study.
